# Risk factors for mortality of diffuse alveolar hemorrhage in systemic lupus erythematosus: a systematic review and meta-analysis

**DOI:** 10.1186/s13075-021-02435-9

**Published:** 2021-02-16

**Authors:** Mengdi Jiang, Ruxuan Chen, Lidan Zhao, Xuan Zhang

**Affiliations:** 1 Department of Rheumatology and Clinical Immunology, Peking Union Medical College Hospital, Chinese Academy of Medical Sciences and Peking Union Medical College, The Ministry of Education Key Laboratory, Beijing, 100730 China; 2 Clinical Immunology Centre, Medical Epigenetics Research Centre, Peking Union Medical College Hospital, Chinese Academy of Medical Sciences and Peking Union Medical College, The Ministry of Education Key Laboratory, Beijing, 100730 China; 3 State Key Laboratory of Difficult, Severe and Rare Diseases, Peking Union Medical College Hospital, Peking Union Medical College and Chinese Academy of Medical Sciences, Beijing, 100730 China

**Keywords:** Systemic lupus erythematosus, Diffuse alveolar hemorrhage, Mortality, Risk factors

## Abstract

**Background:**

Diffuse alveolar hemorrhage (DAH) is a rare but life-threatening complication of systemic lupus erythematosus (SLE). The current knowledge of the prognostic factors for SLE-associated DAH is controversial. This meta-analysis was undertaken to investigate the relevant risk factors for mortality in SLE-associated DAH.

**Methods:**

Studies were searched from PubMed, EMBASE, and Web of Science databases published up to May 27, 2020, and were selected or removed according to the inclusion and exclusion criteria. Two reviewers extracted data independently from the enrolled studies, and the odds ratios (OR) or the standardized mean difference (SMD) was utilized to identify and describe the prognostic factors for mortality.

**Results:**

Eight studies encompassing 251 patients with SLE-associated DAH were included in the meta-analysis. No significant publication bias was shown. Age at the diagnosis of DAH (SMD = 0.35, 95% confidence interval (CI) (0.08, 0.61), *P* = 0.01, *I*^2^ = 0.0%) was found to be an independent risk factor of mortality. Longer lupus disease duration (SMD = 0.28, 95% CI (0.01, 0.55), *P* = 0.042, *I*^2^ = 0.0%), concurrent infection (OR = 2.77, 95% CI (1.55, 4.95), *P* = 0.001, *I*^2^ = 37.5%), plasmapheresis treatment (OR = 1.96, 95% CI (1.04, 3.70), *P* = 0.038, *I*^2^ = 14.6%), and mechanical ventilation (OR = 6.11, 95% CI (3.27, 11.39), *P* < 0.0001, *I*^2^ = 23.3%) were also related to poor survival, whereas no noticeable relationships were revealed between survival and concurrent lupus nephritis (OR = 5.45, 95% CI (0.52, 56.95), *P* = 0.16, *I*^2^ = 58.4%) or treatment of cyclophosphamide (CTX) (OR = 0.74, 95% CI (0.16, 3.41), *P* = 0.70, *I*^2^ = 75.5%).

**Conclusions:**

Older age at the diagnosis of DAH, longer disease duration of SLE, concurrent infection, plasmapheresis treatment, and mechanical ventilation were found related to increased mortality in patients with SLE-associated DAH according to our meta-analysis. However, due to limited studies with heterogeneity, these results should be interpreted cautiously. Notably, severe diseases rendered the requirement of plasmapheresis treatment and mechanical ventilation are themselves associated with poor outcome. Randomized trials of therapeutics are needed to determine the most efficacious strategies for SLE-associated DAH for better management of this life-threatening complication.

**Supplementary Information:**

The online version contains supplementary material available at 10.1186/s13075-021-02435-9.

## Background

Systemic lupus erythematosus (SLE) is a chronic, autoimmune disorder characterized by loss of self-tolerance, systemic inflammation, and multi-organ impairment [1]. Pulmonary involvement associated with SLE commonly presents as pleuritis, acute lupus pneumonia, diffuse alveolar hemorrhage (DAH), and pulmonary arterial hypertension. DAH manifests as shortness of breath with hemoptysis and abrupt drop of hemoglobin levels. The imaging characteristics on computed tomography scans are diffuse patchy ground-glass opacities or hilar-centered consolidation indicating alveolar filling lesion. As a rare but life-threatening complication of SLE, DAH is often devastating with a mortality rate of up to 85.7% as previously reported [2].

Prognostic factors related to the mortality of SLE-associated DAH have been investigated [3–10]. However, published studies present conflicting data on the association of platelet level, systemic lupus erythematosus disease activity index (SLEDAI), and the concurrence of lupus nephritis (LN) with the outcome of SLE-associated DAH [6, 7, 9, 10]. With regard to the SLE-associated DAH treatment paradigms, cyclophosphamide (CTX) therapy was suggested associated with high mortality in one retrospective study [3], whereas it was suggested as protective in another study for SLE-associated DAH [10]. The requirement of mechanical ventilation was proposed as an indicator for poor outcome in several studies [6–9]. Several retrospective studies have investigated this concern and drawn diverse conclusions.

To calibrate personal therapeutic strategies and improve the prognosis of SLE-associated DAH, it is crucial to take prognostic indicators into consideration in routine care. Here, we performed a systematic review and meta-analysis of published observational studies and reviewed the potential prognostic factors for SLE-associated DAH.

## Methods

### Search strategies

This meta-analysis was conducted according to the Preferred Reporting Items for Systematic Reviews and Meta-Analyses (PRISMA) Statement protocol [11].

To investigate the prognostic factors for DAH in patients with SLE, relevant articles were comprehensively and systematically identified from the online databases PubMed, EMBASE, and Web of Science, published up to May 27, 2020. The search strategy consisted of the following keywords in combination with Medical Subject Headings (MeSH) terms in PubMed, Emtree in EMBASE, and text words: (“lupus erythematosus, systemic” or “systemic lupus erythematosus”) and (“alveolar haemorrhage” or “alveolar hemorrhage” or “lung hemorrhage”), and “mortality”.

### Eligibility criteria

Cohort studies and case-control studies that investigated the prognostic factors for DAH in patients with SLE were potentially eligible for the meta-analysis. Specifically, the following criteria were required to be met: (1) studies published in English, (2) patients diagnosed with SLE, (3) patients diagnosed with DAH, and (4) odds ratio (OR) or risk ratio (RR) estimates with 95% confidence interval (CI) and *P* values or values that could be calculated according to the reported data in the study. The major exclusion criteria were as follows: (1) duplicates, reviews, case reports, comments, or editorials; (2) conference abstracts superseded by publication; (3) simple description without comparison; and (4) not related to SLE or DAH.

### Data extraction

Two reviewers (JMD and CRX) independently assessed each study for eligibility based on the inclusion and exclusion criteria and extracted the following data: (1) first author’s surname, (2) publication year, (3) country, (4) study design, (5) number of patients (along with gender), (6) time span, and (7) outcome indicators. Any differences of opinion between the two assessors were discussed until consensus was achieved. When necessary, an additional researcher (ZLD) was contacted for extended discussions as a third adjudicator.

### Quality assessment

Quality assessment of each study was performed independently by the two authors (JMD and CRX), using the Newcastle-Ottawa Scale (NOS) (http://www.ohri.ca/programs/clinical_epidemiol-ogy/oxford.asp), which graded studies according to the quality of selection, comparability, and outcome of study participants. The NOS utilizes a score rating system (with scores ranging from 0 to 9) to evaluate the quality of each study. Studies awarded six or more scores are regarded as high quality. Discrepancies were addressed by the re-evaluation of the original article and discussion with a third independent reviewer.

### Statistical analysis

All statistical analyses were carried out using the STATA 14 software (Stata Corporation, College Station, TX, USA). OR was evaluated for counting data, and the standardized mean difference (SMD) was measured for the measurement data. OR and RR estimates were considered equivalent in the meta-analysis given the extremely low incidence of SLE-DAH. Heterogeneity was calculated using Higgins’s (*I*^2^) test statistic. A value of *I*^2^ of 0–25% represents no heterogeneity, 26–50% represents low heterogeneity, 51–75% represents moderate heterogeneity, and > 75% represents high heterogeneity [12]. If significant heterogeneity (*P* < 0.10 or *I*^2^ > 50%) existed, the random-effects model was applied. Otherwise, the fixed-effects model was utilized. In order to assess the stability and reliability of the results, a sensitivity analysis was performed by sequential omission of individual studies. In addition, funnel plot and Begg’s test were used to evaluate publication bias [13]. *P* value less than 0.05 was considered significant.

## Results

### Characteristics of enrolled studies

**Fig. 1:**
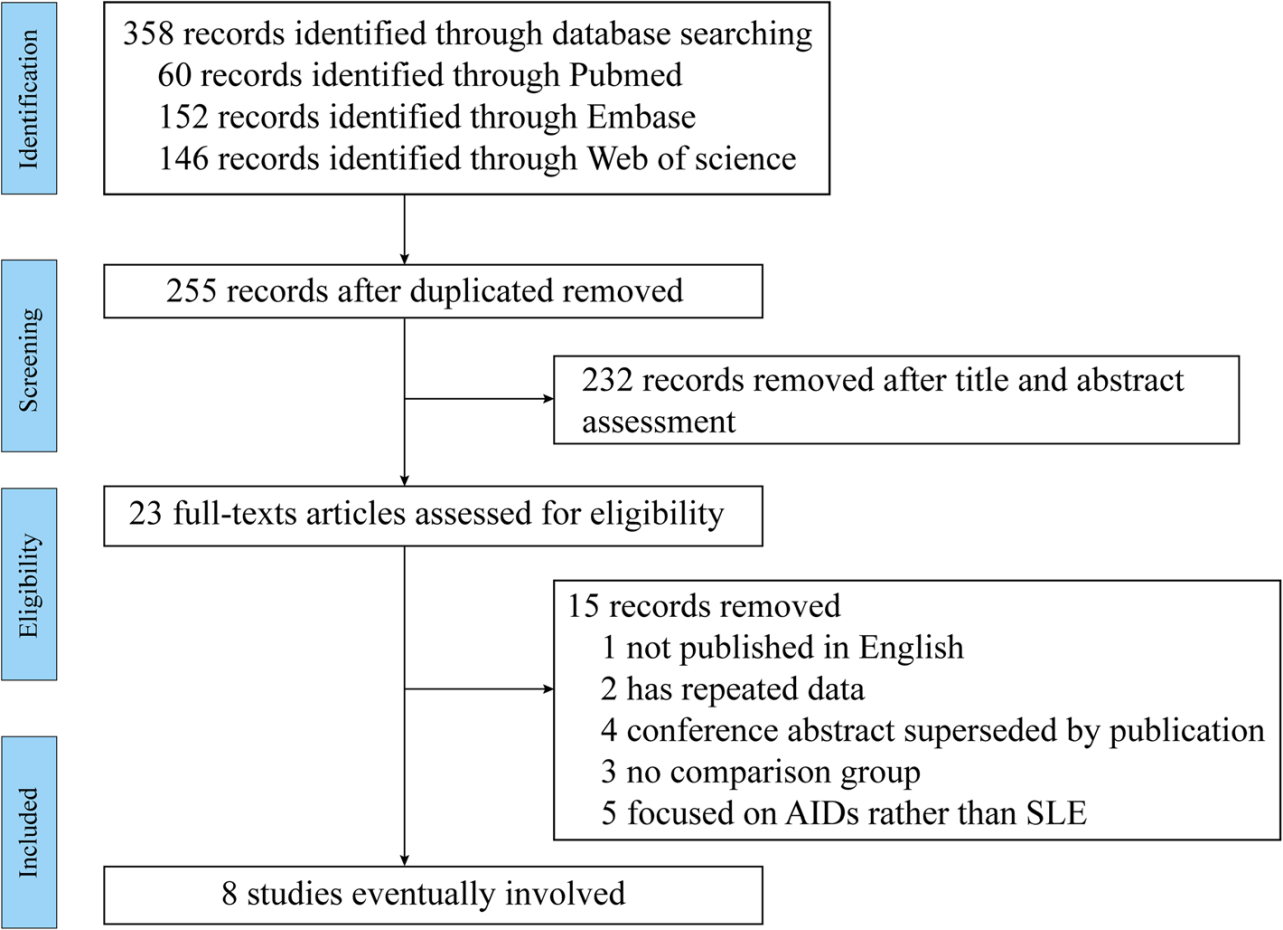
Flowchart of the literature selection process

A total of 358 potentially eligible articles were obtained from the initial retrieval, of which 103 were excluded due to duplicate publication. After title and abstract screening, 232 articles unrelated to SLE-associated DAH were excluded, then the 23 remaining articles were reviewed for full texts. Fifteen articles were excluded according to the exclusion and inclusion criteria. Finally, 8 studies including 251 SLE patients with 262 episodes of DAH were selected, and data items were extracted. A flowchart of article screening for the meta-analysis is illustrated in Fig. 1.

All these patients fulfilled the revised criteria of the American College of Rheumatology for classification of SLE [14, 15]. The included 8 studies employed the same diagnostic criteria for DAH including (1) one or more pulmonary symptoms or signs including dyspnea, hypoxemia, hemoptysis, or cough; (2) acute diffuse lung infiltration on radiological images (chest X-ray or computed tomography) suggestive of alveolar hemorrhage; (3) a sudden drop in hemoglobin levels of ≥ 1.5 g/dL in the absence of bleeding elsewhere; and (4) the presence of hemosiderin-laden macrophages on bronchoalveolar lavage or lung biopsy demonstrating DAH.

All 8 studies compared the mortality risk factors including demographic features, clinical characteristics, laboratory data, and comorbidity between deceased and survivor groups. The characteristics of the eight included studies are summarized in Table 1. These studies were carried out in South Korea (*n* = 2), China (*n* = 2), Mexico (*n* = 1), the USA (*n* = 1), Colombia (*n* = 1), and Singapore (*n* = 1). The methodological quality of the included studies was relatively high according to the 3 items (selection, comparability on the basis of the design or analysis, and outcome) considered in the Newcastle-Ottawa Scale (Additional file 1).

**Table 1:** Baseline characteristics of patients in the included studies

No.	Author, year	Country	Study population	Study type	Time span	Number of patients (gender)	Prognostic factors	Mortality rate (%)	NOS score
1	Zamora, 1997	USA	SLE patients from a single cohort	Retrospective cohort study	1984–1994	15 (10 females, 5 male)	CTX treatment, mechanical ventilator, infection	42.1	8
2	Chang, 2002	China	SLE patients from a single cohort	Retrospective cohort study	1994–2001	8 (all females)	APACHE II score, OSF score	50.0	8
3	Badsha, 2004	Singapore	SLE patients from a single cohort	Retrospective cohort study	1994–2001	22 (20 females, 2 males)	SLEDAI, SLAM	36.4	8
4	Kwok, 2011	South Korea	SLE patients from a single cohort	Retrospective cohort study	1993–2009	21 (19 females, 2 males)	Mechanical ventilator, infection	61.9	9
5	Martínez-Martínez, 2014	Mexico	SLE patients from 9 cohorts	Retrospective cohort study	NA	50 patients with 57 episodes (49 females, 8 males)	Creatinine, platelets, APACHE II score, mechanical ventilator, infection	42.1	8
6	Kim, 2017	South Korea	SLE patients from a single cohort	Retrospective cohort study	2004–2014	24 (all females)	Lupus nephritis, plasmapheresis, transfusion, mechanical ventilator	29.2	9
7	Quintana, 2019	Colombia	SLE patients from a single cohort	Retrospective cohort study	2011–2018	17 (11 females, 6males)	Disease duration, albumin, SLEDAI, inotropic/vasoactive use, mechanical ventilator	29.4	7
8	Sun, 2020	China	SLE patients from a single cohort	Retrospective cohort study	2004–2019	94 (82 females, 12 males)	Lupus nephritis, platelets, CTX treatment, mechanical ventilator	38.3	9

*NOS* Newcastle-Ottawa Scale, *SLE* systemic lupus erythematosus, *CTX* cyclophosphamide, *APACHE II* Acute Physiology, Age, and Chronic Health Evaluation, *OSF* organ system failure, *SLEDAI* systemic lupus erythematosus activity index, *SLAM* systemic lupus activity measure, *NA* not available

### Prognostic factors for survival of SLE-associated DAH patients

#### Comparison of demographic and clinical characteristics

**Fig. 2:**
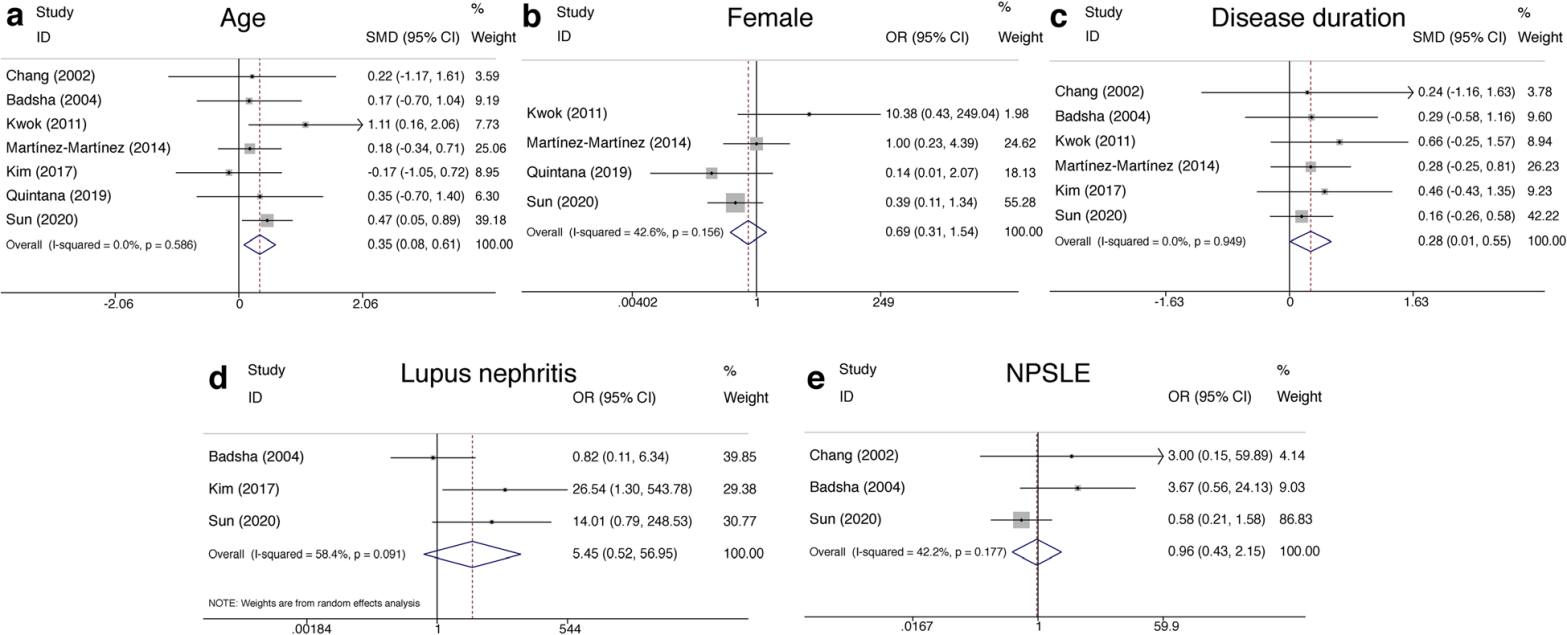
Forest plots of demographic features and clinical characteristics in SLE-associated DAH. **a** Age. **b** The female group. **c** Disease duration. **d** Lupus nephritis. **e** NPSLE. SLE, systemic lupus erythematosus; DAH, diffuse alveolar hemorrhage; OR, odds ratios; SMD, standardized mean difference; NPSLE, neuropsychiatric lupus erythematosus

Seven studies with no heterogeneity compared the age at diagnosis between the deceased and the survivors in SLE-associated DAH patients utilizing the fixed-effects model (*P* = 0.59, *I*^2^ = 0.0%). The pooled data indicated a significant correlation of older ages at diagnosis with the poor outcome (SMD = 0.35, 95% CI (0.08, 0.61), *P* = 0.01) (Fig. 2a, Table 2). The association between gender and the survival of patients with SLE-associated DAH was non-remarkable (OR = 0.69, 95% CI (0.31, 1.54), *P* = 0.369) (Fig. 2b, Table 2). For disease duration, six studies containing the data of disease duration were pooled, and the analysis showed that this factor was correlated with mortality employing the fixed-effects model (SMD = 0.28, 95% CI (0.01, 0.55), *P* = 0.042) (Fig. 2c, Table 2).

**Table 2:** Meta-analysis results for included studies of the relationship between risk factors and mortality in SLE-associated DAH patients

Variables	No. of studies	Number of enrolled episodes	Effects model	*I*^2^ (%)	OR/SMD (95%CI)	*P* values	Relationship	Publication bias (*P* value of Begg’s test)
Demographic and clinical characteristics								
Age	7	243	Fixed	0.0%	0.35 (0.08, 0.61)	0.010	Increased risks	None (1.000)
Female	4	189	Fixed	42.6%	0.69 (0.31, 1.54)	0.369	No association	None (0.734)
Disease duration	6	226	Fixed	0.0%	0.28 (0.01, 0.55)	0.042	Increased risks	None (0.260)
Lupus nephritis	3	140	Random	58.4%	5.45 (0.52, 56.95)	0.160	No association	None (0.296)
NPSLE	3	124	Fixed	42.2%	0.96 (0.43, 2.15)	0.920	No association	None (1.000)
Laboratory data and disease activity								
Platelet	6	219	Random	69.1%	− 0.29 (− 0.86, 0.29)	0.328	No association	None (0.260)
Drop of hemoglobin	5	202	Random	57.5%	0.34 (− 0.17, 0.85)	0.190	No association	None (0.806)
C3	3	132	Random	77.0%	0.48 (− 0.51, 1.46)	0.340	No association	None (1.000)
SLEDAI	6	186	Random	76.9%	0.24 (− 0.52, 0.99)	0.540	No association	None (1.000)
Comorbidity and treatment								
Infection	6	223	Fixed	37.5%	2.77 (1.55, 4.95)	0.001	Increased risks	None (0.260)
CTX treatment	6	224	Random	75.5%	0.74 (0.16, 3.41)	0.700	No association	None (1.000)
IVIG treatment	3	175	Random	61.2%	1.28 (0.24, 6.87)	0.780	No association	None (0.296)
Plasmapheresis	5	167	Fixed	14.6%	1.96 (1.04, 3.70)	0.038	Increased risks	None (0.806)
Mechanical ventilation	8	262	Fixed	23.3%	6.11 (3.27, 11.39)	< 0.0001	Increased risks	None (1.000)

*SLE* systemic lupus erythematosus, *DAH* diffuse alveolar hemorrhage, *OR* odds ratios, *SMD* standardized mean difference, *NPSLE* neuropsychiatric lupus erythematosus, *C3* complement 3, *SLEDAI* systemic lupus erythematosus disease activity index, *CTX* cyclophosphamide, *IVIG* intravenous immunoglobulin

With regard to concomitant diseases, three studies investigated and measured the correlation between the occurrence of LN or neuropsychiatric lupus erythematosus (NPSLE) with the survival of SLE-associated DAH. The pooled data indicated no statistically difference in the presence of nephritis or NPSLE in the deceased group compared with the survivor group (OR = 5.45, 95% CI (0.52, 56.95), *P* = 0.16 and OR = 0.96, 95% CI (0.43, 2.15), *P* = 0.92) (Fig. 2d, e, Table 2).

#### Comparison of laboratory data and disease activity

**Fig. 3:**
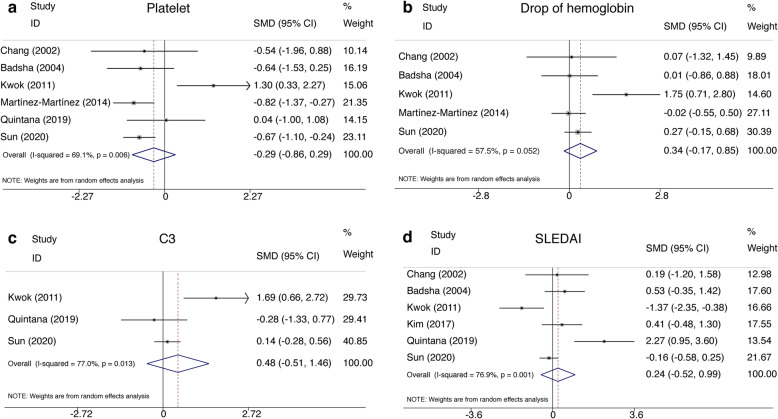
Forest plots of laboratory data and disease activity in SLE-associated DAH. **a** The level of platelet. **b** Drop of hemoglobin. **c** C3. **d** SLEDAI. SLE, systemic lupus erythematosus; DAH, diffuse alveolar hemorrhage; SMD, standardized mean difference; C3, complement 3; SLEDAI, systemic lupus erythematosus disease activity index

When referring to relevant laboratory indices, six studies were gathered to evaluate the correlation between the levels of platelet and the survival of SLE-associated DAH patients using the random-effects model with moderate heterogeneity among studies (*P* = 0.01, *I*^2^ = 69.1%). The pooled data indicated no significant difference in levels of platelet between the deceased group and the survivor group (SMD = − 0.29, 95% CI (− 0.86, 0.29), *P* = 0.328) (Fig. 3a, Table 2). In addition, by utilizing random-effects model, our pooled results revealed that neither the drop of hemoglobin nor the level of complement 3 (C3) during DAH episodes was related to the mortality in SLE-associated DAH patients (SMD = 0.34, 95% CI (− 0.17, 0.85), *P* = 0.19 and SMD = 0.48, 95% CI (− 0.51, 1.46), *P* = 0.34) (Fig. 3b, c, Table 2).

A total of six studies with high heterogeneity compared the average SLEDAI score between the deceased and the survivors in SLE-associated DAH patients. By utilizing the random-effects model (*P* = 0.001, *I*^2^ = 76.9%), the pooled data showed the SLEDAI score was not relevant to the outcome (SMD = 0.24, 95% CI (− 0.52, 0.99), *P* = 0.54) (Fig. 3d, Table 2).

#### Comparison of comorbidity and treatment

**Fig. 4:**
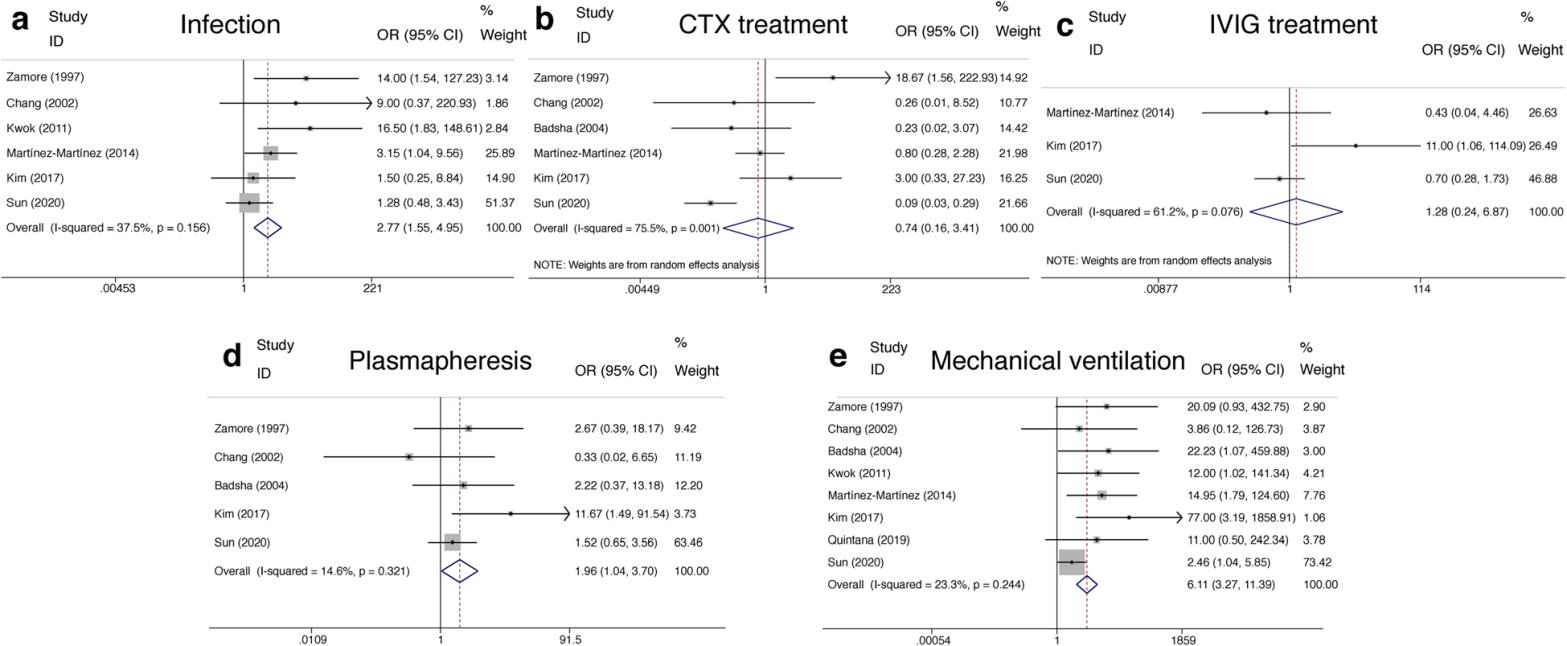
Forest plots of comorbidity and treatment in SLE-associated DAH. **a** Infection. **b** CTX treatment. **c** IVIG treatment. **d** Plasmapheresis. **e** Mechanical ventilation. SLE, systemic lupus erythematosus; DAH, diffuse alveolar hemorrhage; OR, odds ratios; CTX, cyclophosphamide; IVIG, intravenous immunoglobulin

Infection, as a threatening comorbidity of DAH, was analyzed in 6 studies with low heterogeneity. To determine the relationship of infection with survival, the fixed-effects model (*P* = 0.16, *I*^2^ = 37.5%) was used to analyze the pooled data, and a significant higher incidence of infection in the deceased group than the survivor group was revealed (OR = 2.77, 95% CI (1.55, 4.95), *P* = 0.001) (Fig. 4a, Table 2).

With regard to the treatment, based on the random-effects model, the associations between the survival of SLE-associated DAH and the treatment of CTX or intravenous immunoglobulin (IVIG) were found non-remarkable (OR = 0.74, 95% CI (0.16, 3.41), *P* = 0.70 and OR = 1.28, 95% CI (0.24, 6.87), *P* = 0.78) (Fig. 4b, c, Table 2). Further, five studies with no heterogeneity were pooled for evaluating the relationship of plasmapheresis and survival. Surprisingly, a higher rate of plasmapheresis application was revealed in the deceased group compared with the survivor group (OR = 1.96, 95% CI (1.04, 3.70), *P* = 0.038) (Fig. 4d, Table 2). Finally, mechanical ventilation as a rescue support for hypoxemia which could develop from severe DAH had been assessed in a total of 8 studies, and the pooled data revealed a higher frequency of mechanical ventilation in the deceased group than in the survivor group using the fixed-effects model (OR = 6.11, 95% CI (3.27, 11.39), *P* < 0.0001) (Fig. 4e, Table 2).

### Sensitivity analysis

Sensitivity analysis was conducted by deleting a single study each time to observe the influence of each study on the overall analysis of outcome. For the level of platelet, sensitivity analysis suggested that “Kwok 2011” was the primary cause of the heterogeneity for the pooled data (*I*^2^ = 67% vs. *I*^2^ = 0.0%), and omitting this study changed the significance of the pooled results (*P* = 0.32 vs. *P* < 0.0001) (Additional file 2). For the occurrence of LN, sensitivity analysis suggested that “Badsha 2004” was the primary cause of the heterogeneity for the pooled data (*I*^2^ = 58% vs. *I*^2^ = 0.0%), and omitting this study changed the significance of the pooled results (*P* = 0.16 vs. *P* = 0.006).

### Publication bias

Funnel plot and Begg’s test were used to evaluate publication bias. There was no obvious funnel plot asymmetry (Additional file 3). The *P* value of Begg’s test was > 0.05, indicating that publication bias was not evident in our meta-analysis.

## Discussion

As a catastrophic clinical syndrome capable of inducing respiratory failure, DAH has various etiology such as infections, systemic autoimmune diseases, transplantation, and medicine [16]. DAH occurs in 0.6–5.7% of patients with SLE admitted to the hospital and may present as the initial manifestation in 10–20% of cases [3, 17, 18]. Pulmonary capillaritis is considered as the underlying pathology. The deposition of immune complexes and the activation of complement within the lungs are central to the development of parenchymal disease in SLE [19]. The reported mortality of SLE-associated DAH was as high as 85.7%, and cohort studies have been carried out to investigate the risk factors for poor outcome, but the results were inconsistent and no agreement has been reached so far. In this study, we conducted a meta-analysis to estimate the prognostic factors of SLE-associated DAH. Although studies meeting the strict eligibility criteria were limited and heterogeneity existed, this is the first study providing comprehensive information on the survival of patients with SLE-associated DAH based on current prognostic cohort studies. In this meta-analysis with pooled data, older age at DAH occurrence, longer lupus disease duration, application of mechanical ventilation, and plasmapheresis utilization were found associated with mortality, whereas no noticeable relationships were revealed between survival and gender, concurrent LN, or treatment of CTX or IVIG.

It has been reported that a 10-year survival rate of late-onset SLE was poorer than that of early-onset SLE [20], indicating the possible contribution of age at diagnosis for mortality. We found a statistically significant difference with respect to age in the pooled data, with an older age at diagnosis in the deceased group on average than in those who survived. Additionally, our study suggested that longer lupus disease duration was associated with increased mortality while gender was not. Three enrolled studies addressed concurrent LN. LN, often presenting as acute glomerulonephritis, when complicated with DAH, can be considered as pulmonary renal vasculitis syndrome which heralds the requirement of urgent, aggressive therapy [21]. LN is used to be considered a poor prognostic factor for SLE [8], but our pooled data revealed no noticeable relationships between LN and poor survival in SLE-DAH. Explanations could be that DAH itself can lead to high mortality, far faster exceeding what LN can contribute; thus, the “add-on” of LN makes no difference. Another possibility is that limited numbers of studies and relatively small sample size have brought bias. Notably, though sensitivity analysis suggested that excluding “Badsha 2004,” a primary cause of the heterogeneity for the pooled data, could change the significance of LN in the pooled results (*P* = 0.16 vs. *P* = 0.006), deletion of this study may reduce the degree of confidence with only two remaining studies enrolled. More studies and a larger sample size concerning this issue may help identify the role of LN in the outcome of SLE-DAH.

Severe thrombocytopenia can cause bleeding, aggravating hypoxemia and blood-loss anemia in DAH. Thrombocytopenia has been identified as a harmful prognostic factor in several enrolled studies [7, 10], but the pooled data did not reach the same conclusion and the level of platelet seemed unrelated to mortality. Notably, sensitivity analysis suggested that one of the pooled studies, “Kwok 2011,” was the primary source of the heterogeneity in pooled data (*I*^2^ = 67% vs. *I*^2^ = 0.0%), and excluding this single study changed the significance of the pooled results (*P* = 0.32 vs. *P* < 0.0001). Nonetheless, in studies grading according to the quality of selection, comparability, and outcome of study participants, the Newcastle-Ottawa Assessment Scale of “Kwok 2011” turned out to be excellent so as not removed from the pooled analysis. The small scale of this study may account for this divergent laboratory data. Further, large-scale prognostic studies are warranted to identify the contribution of platelet to the outcome of DAH. Acute drop of hemoglobin may indicate severe disease progression of DAH but may not always match due to possible confounders like hemolytic anemia and inflammation-associated anemia [10]. Similar with the conclusions proposed by most cohorts [4–7, 10], our study suggested that the drop of hemoglobin was not a prognostic factor for mortality. However, without specific information about the baseline hemoglobin and the time span for the drop of hemoglobin in these cohorts, this result should be interpreted cautiously. Interestingly, SLEDAI and C3, related to SLE activity, were not identified as predictors of mortality in SLE-associated DAH cohorts. However, due to the significant heterogeneity of these factors, further verifications are needed.

Due to the potent immunosuppressive drugs and immune disturbances of lupus itself, infection is very common in patients with SLE and is regarded as the top cause for mortality in SLE [22, 23]. Blood, filled in the alveolar spaces in a patient with DAH, is a favorable medium for bacteria growth, which increases the risk of infection. In addition, infection will complicate the situation of DAH and make treatment more formidable. As shown in our study, infection was a crucial and fatal factor for prognosis in SLE-associated DAH. Thus, prevention, detection, differentiation and elimination of infections in the whole course of DAH are of essential importance. This underscores the necessity of evaluation of the risk-benefit of intensive immunosuppressive therapy and justifies anti-infection treatment whenever there is a suspicion. Not surprisingly, mechanical ventilation has been suggested to associate with increased mortality in SLE-associated DAH [5–8, 10]. Though mechanical ventilation can increase the risk of iatrogenic injury, the requirement of mechanical ventilation itself is usually the sign of most severe illness in clinical practice. The association between mechanical ventilation and the outcomes is unlikely causative, instead, may just reflect the disease severity.

No prospective randomized controlled trials have been implemented to verify the association of treatment modalities and the outcome of DAH in SLE. Nevertheless, high doses of glucocorticoids are generally accepted as the cornerstone in treating SLE-associated DAH for decades [24]. As previously reported in cohort studies, both the “usage of intravenous glucocorticoids” and “pulse of methylprednisolone” have been analyzed as non-significant on survival [5, 8, 10]. The wide-spread application of glucocorticoids in patients with DAH may account for this. Furthermore, Barile et al. once suggested that patients who received aggressive pulses of methylprednisolone (4–8 g of total dose) had a better survival than those with conventional pulses of methylprednisolone (3 g in total) [25].

Cyclophosphamide, an immunosuppressor commonly suggested in severe lupus, was reported being used in approximately half of the lupus patients with DAH [26]. However, the association of CTX with survival hasn’t been confirmed in this meta-analysis. Sensitivity analysis revealed that this pooled survival data was stable. But the contradictory conclusions have been given in different studies with an unfavorable effect of CTX on survival suggested by one early study (e.g., Zamora et al. [3]), in contrast to the favorable effect of CTX suggested by a recent study (e.g., Sun et al. [10])*.* The advances in the care of critically ill patients and better control of CTX utilization in recent years may explain the conflicting results [26].

Plasmapheresis can remove pathogenic autoantibodies and immune complexes from circulation [27] and was used in about 5.9~66.6% SLE-associated DAH as reported [18]. However, unlike the agreement on its definite efficacy in Goodpasture’s syndrome [28], its efficacy in patients with SLE-associated DAH is controversial. Although one case-series study once demonstrated plasmapheresis as an effective treatment in DAH in the 1990s [29], later increasing studies indicated that plasmapheresis did not improve survival [3, 6, 10]. The authors speculated the lack of efficacy of plasmapheresis could be either because autoantibodies and immune complex may not the main etiology of perpetuating DAH or because the pathogenic antibodies have bound to the alveolar membrane and precipitated in tissue thus unremovable by this treatment [26]. Subsequently, Kim et al. even found that plasmapheresis could be a risk factor for poor outcome in SLE-associated DAH patients [8]. Notably, in clinical practice, plasmapheresis is more constantly applied to patients with refractory or severe diseases [17, 29], especially in those who inadequately respond to a high-dose pulse of corticosteroid and cyclophosphamide therapy [30], which may itself relate to poor survival. Still, rheumatologists should be reminded of the appropriate usage of plasmapheresis. According to the registry of the World Apheresis Association, in patients with autoimmune diseases, the rate of adverse events of apheresis could reach more than 10% [31]. And related complications derived from invasive procedures such as bleeding or catheter-related infections should be monitored and tightly controlled [32].

Several limitations existed in this study. So far, prospective random controlled studies addressing this issue are lacking, so enrolled studies are primarily of retrospective design. The heterogeneity across enrolled studies is another limitation, which partly derived from lupus heterogeneity itself and partly derived from the small scale of this study. Due to limited numbers of studies and insufficient information provided by the original articles, subgroup analyses are not performed to trace the heterogeneity origin. Sensitivity analysis suggested some particular study like “Kwok 2011” could change the significance of the pooled results on the level of platelet, alerting us of possible bias. Moreover, we are unable to investigate the influence of glucocorticoids usage on survival with pooled data, due to the various evaluation methods of its usage in enrolled studies. A larger scale of studies and more investigations are needed for identifying the prognostic indicators of SLE-associated DAH in the future. Also, randomized trials are warranted to identify the benefits of aggressive therapies like plasmapheresis and to determine the most efficacious strategies.

## Conclusions

DAH is a rare but life-threatening complication of SLE. Our meta-analysis suggested that older age at DAH diagnosis, longer lupus disease duration, concurrent infection, plasmapheresis treatment, and the application of mechanical ventilation were related to poor survival in patients with SLE-associated DAH, which should attract greater clinical attention and be taken into consideration for treatment calibration.

## Supplementary Information

**Additional file 1.** : Newcastle-Ottawa Quality Assessment Scale for the meta-analysis.

**Additional file 2 **: Sensitivity analysis of each prognostic factor included study. **a**. age; **b.** the female group; **c.** disease duration. **d**. lupus nephritis; **e.** NPSLE; **f.** the level of platelet; **g.** drop of hemoglobin; **h.** C3; **i.** SLEDAI; **j.** infection; **k.** CTX treatment; **l.** IVIG treatment; **m.** plasmapheresis; **n.** mechanical ventilation; NPSLE: neuropsychiatric lupus erythematosus; C3: complement 3; SLEDAI: systemic lupus erythematosus disease activity index; CTX: cyclophosphamide; IVIG: intravenous immunoglobulin.

**Additional file 3 **: Begg’s funnel plots of the publication bias. **a**. age; **b.** the female group; **c.** disease duration. **d**. lupus nephritis; **e.** NPSLE; **f.** the level of platelet; **g.** drop of hemoglobin; **h.** C3; **i.** SLEDAI; **j.** infection; **k.** CTX treatment; **l.** IVIG treatment; **m.** plasmapheresis; **n.** mechanical ventilation; NPSLE: neuropsychiatric lupus erythematosus; C3: complement 3; SLEDAI: systemic lupus erythematosus disease activity index; CTX: cyclophosphamide; IVIG: intravenous immunoglobulin.

## Data Availability

Data sharing is not applicable to this article as no datasets were generated or analyzed during the current study.

## References

[1] Tsokos GC (2011). Systemic lupus erythematosus. N Engl J Med.

[2] Mintz G, Galindo LF, Fernández-Diez J, Jiménez FJ, Robles-Saavedra E, Enríquez-Casillas RD (1978). Acute massive pulmonary hemorrhage in systemic lupus erythematosus. J Rheumatol.

[3] Zamora MR, Warner ML, Tuder R, Schwarz MI (1997). Diffuse alveolar hemorrhage and systemic lupus erythematosus - clinical presentation, histology, survival, and outcome. Medicine..

[4] Chang MY, Fang JT, Chen YC, Huang CC (2002). Diffuse alveolar hemorrhage in systemic lupus erythematosus: a single center retrospective study in Taiwan. Ren Fail.

[5] Badsha H, Cheng LT, Kok OK, Tsui YL, Hiok HC (2004). Pulmonary hemorrhage in systemic lupus erythematosus. Semin Arthritis Rheum.

[6] Kwok SK, Moon SJ, Ju JH, Park KS, Kim WU, Cho CS (2011). Diffuse alveolar hemorrhage in systemic lupus erythematosus: risk factors and clinical outcome: results from affiliated hospitals of Catholic University of Korea. Lupus..

[7] Martinez-Martinez MU, Sturbaum AK, Alcocer-Varela J, Merayo-Chalico J, Gómez-Martin D, Gómez-Bañuelos Jde J (2014). Factors associated with mortality and infections in patients with systemic lupus erythematosus with diffuse alveolar hemorrhage. J Rheumatol.

[8] Kim D, Choi J, Cho SK, Choi CB, Kim TH, Jun JB (2017). Clinical characteristics and outcomes of diffuse alveolar hemorrhage in patients with systemic lupus erythematosus. Semin Arthritis Rheum.

[9] Quintana JH, Aragon CC, Santos V-A, de Las Salas A, Tafur R-A, Aguirre-Valencia D, et al. Diffuse alveolar hemorrhage: a cohort of patients With Systemic Lupus Erythematosus. J Clin Rheumatol. 2020;26(7S Suppl 2):S153–7. 10.1097/RHU.0000000000001228 31895107

[10] Sun Y, Zhou C, Zhao J, Wang Q, Xu D, Zhang S, et al. Systemic lupus erythematosus-associated diffuse alveolar hemorrhage: a single-center, matched case-control study in China. Lupus. 2020;29(7):795–803. 10.1177/0961203320920715 32321345

[11] Moher D, Shamseer L, Clarke M, Ghersi D, Liberati A, Petticrew M (2015). Preferred Reporting Items for Systematic Review and Meta-Analysis Protocols (PRISMA-P) 2015 statement. Syst Rev.

[12] Higgins JP, Thompson SG, Deeks JJ, Altman DG (2003). Measuring inconsistency in meta-analyses. BMJ..

[13] Egger M, Davey Smith G, Schneider M, Minder C (1997). Bias in meta-analysis detected by a simple, graphical test. BMJ..

[14] Tan EM, Cohen AS, Fries JF, Masi AT, McShane DJ, Rothfield NF (1982). The 1982 revised criteria for the classification of systemic lupus erythematosus. Arthritis Rheum.

[15] Hochberg MC (1997). Updating the American College of Rheumatology revised criteria for the classification of systemic lupus erythematosus. Arthritis Rheum.

[16] Lara AR, Schwarz MI (2010). Diffuse alveolar hemorrhage. Chest..

[17] Cañas C, Tobón GJ, Granados M, Fernández L (2007). Diffuse alveolar hemorrhage in Colombian patients with systemic lupus erythematosus. Clin Rheumatol.

[18] Martinez-Martinez MU, Abud-Mendoza C (2014). Diffuse alveolar hemorrhage in patients with systemic lupus erythematosus. Clinical manifestations, treatment, and prognosis. Reumatol Clin.

[19] Churg A, Franklin W, Chan KL, Kopp E, Carrington CB (1980). Pulmonary hemorrhage and immune-complex deposition in the lung. Complications in a patient with systemic lupus erythematosus. Arch Pathol Lab Med.

[20] Merola JF, Bermas B, Lu B, Karlson EW, Massarotti E, Schur PH (2014). Clinical manifestations and survival among adults with (SLE) according to age at diagnosis. Lupus..

[21] Lee RW, D’Cruz DP (2010). Pulmonary renal vasculitis syndromes. Autoimmun Rev.

[22] Sciascia S, Ceberio L, Garcia-Fernandez C, Roccatello D, Karim Y, Cuadrado MJ (2012). Systemic lupus erythematosus and infections: clinical importance of conventional and upcoming biomarkers. Autoimmun Rev.

[23] Kedves M, Kósa F, Kunovszki P, Takács P, Szabó MZ, Karyekar C, et al. Large-scale mortality gap between SLE and control population is associated with increased infection-related mortality in lupus. Rheumatology (Oxford). 2020;59(11):3443–51. 10.1093/rheumatology/keaa188 PMC7590419 32357240

[24] Martínez-Martínez MU, Oostdam DAH, Abud-Mendoza C (2017). Diffuse alveolar hemorrhage in autoimmune diseases. Curr Rheumatol Rep.

[25] Barile LA, Jara LJ, Medina-Rodriguez F, García-Figueroa JL, Miranda-Limón JM (1997). Pulmonary hemorrhage in systemic lupus erythematosus. Lupus..

[26] Ednalino C, Yip J, Carsons SE (2015). Systematic review of diffuse alveolar hemorrhage in systemic lupus erythematosus: focus on outcome and therapy. J Clin Rheumatol.

[27] Kronbichler A, Brezina B, Quintana LF, Jayne DR (2016). Efficacy of plasma exchange and immunoadsorption in systemic lupus erythematosus and antiphospholipid syndrome: a systematic review. Autoimmun Rev.

[28] Pusey CD (2003). Anti-glomerular basement membrane disease. Kidney Int.

[29] Erickson RW, Franklin WA, Emlen W (1994). Treatment of hemorrhagic lupus pneumonitis with plasmapheresis. Semin Arthritis Rheum.

[30] Claridge S, Das P, Dorling A, Robson MG. Plasmapheresis as rescue therapy for systemic lupus erthyematosus-associated diffuse alveolar haemorrhage. BMJ Case Rep. 2011;2011:bcr0220113893. 10.1136/bcr.02.2011.3893. 10.1136/bcr.02.2011.3893 PMC3063259 22698899

[31] Mörtzell Henriksson M, Newman E, Witt V, Derfler K, Leitner G, Eloot S (2016). Adverse events in apheresis: an update of the WAA registry data. Transfus Apher Sci.

[32] Ahmed S, Kaplan A. Therapeutic plasma exchange using membrane plasma separation. Clin J Am Soc Nephrol. 2020;15(9):1364–70. 10.2215/CJN.12501019 PMC7480555 32312791

