# Supplementary material for: Risk factors for mortality of diffuse alveolar hemorrhage in systemic lupus erythematosus: a systematic review and meta-analysis
Source: Arthritis Res Ther. 2021 Feb 16;23:57. doi: 10.1186/s13075-021-02435-9 (PMC7885396; doi:10.1186/s13075-021-02435-9)
Supplement: Supplementary file 1 — Additional file 1. : Newcastle-Ottawa Quality Assessment Scale for the meta-analysis. [file 13075_2021_2435_MOESM1_ESM.docx]

**Additional file 1.** Newcastle-Ottawa Quality Assessment Scale for the meta-analysis

|  | Zamore, 1997 | Chang, 2002 | Badsha, 2004 | Kwok, 2011 | Martínez-Martínez, 2014 | Kim, 2017 | Quintana, 2019 | Sun, 2020 |
| --- | --- | --- | --- | --- | --- | --- | --- | --- |
| **Selection** |  |  |  |  |  |  |  |  |
| Representativeness of the exposed cohort | 1 | 1 | 1 | 1 | 1 | 1 | 1 | 1 |
| Selection of the non-exposed cohort | 1 | 1 | 1 | 1 | 1 | 1 | 1 | 1 |
| Ascertainment of exposure | 1 | 1 | 1 | 1 | 1 | 1 | 1 | 1 |
| Demonstration that outcome of interest was not present at start of study | 1 | 1 | 1 | 1 | 1 |  |  |  |
| **Comparability** | 1 | 1 | 1 | 2 | 2 | 2 | 1 | 2 |
| **Outcome** |  |  |  |  |  |  |  |  |
| Assessment of outcome | 1 | 1 | 1 | 1 | 1 | 1 | 1 | 1 |
| Was follow-up long enough for outcomes to occur | 1 | 1 | 1 | 1 | 1 | 1 | 1 | 1 |
| Adequacy of follow-up of cohort | 1 | 1 | 1 | 1 | 0 | 1 | 0 | 1 |
| **Total score** | 8 | 8 | 8 | 9 | 8 | 9 | 7 | 9 |
