# Supplementary figures and images for: Risk factors for mortality of diffuse alveolar hemorrhage in systemic lupus erythematosus: a systematic review and meta-analysis
Source: Arthritis Res Ther. 2021 Feb 16;23:57. doi: 10.1186/s13075-021-02435-9 (PMC7885396; doi:10.1186/s13075-021-02435-9)

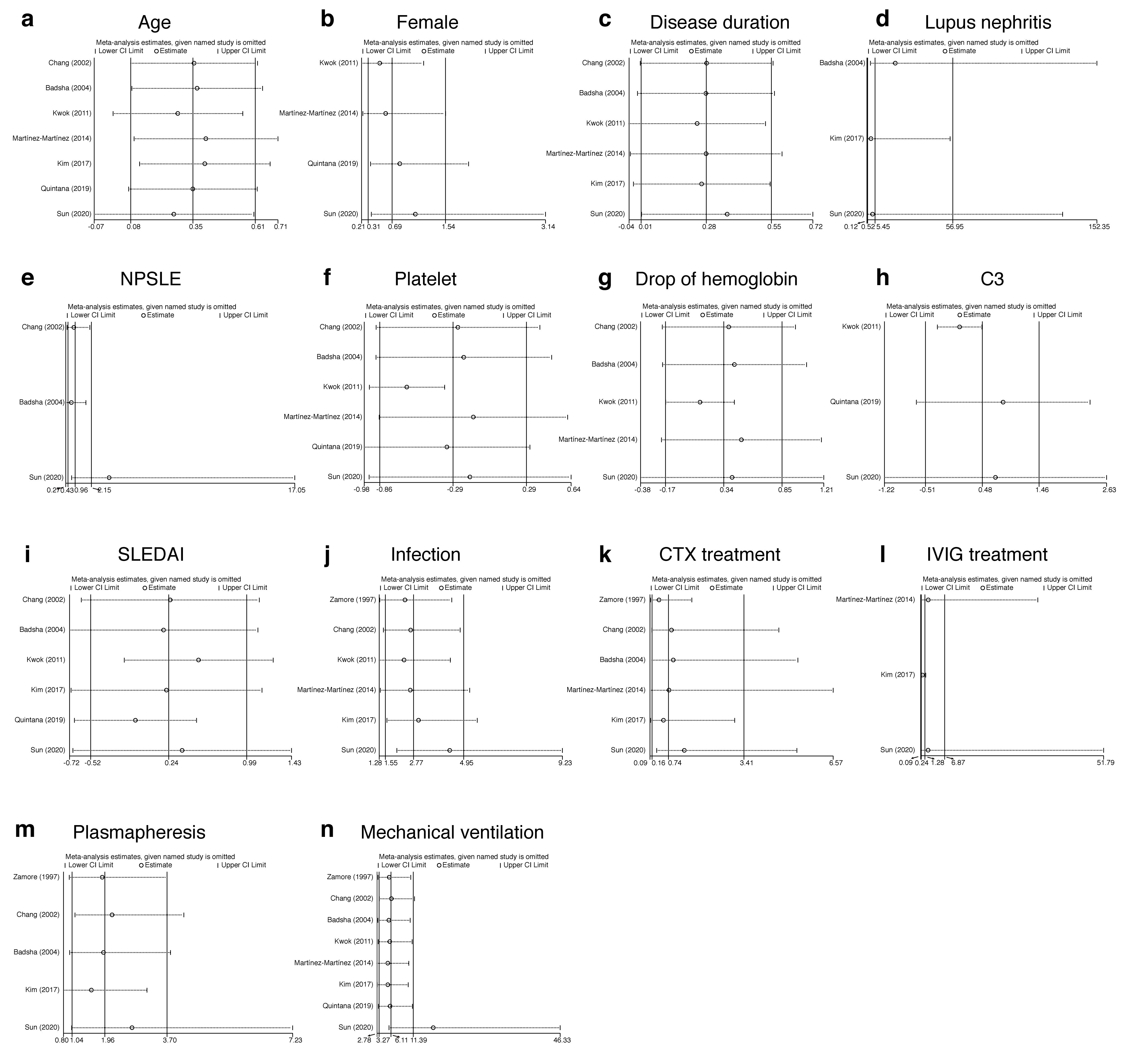

Supplement: Supplementary file 2 — Additional file 2 : Sensitivity analysis of each prognostic factor included study. a. age; b. the female group; c. disease duration. d. lupus nephritis; e. NPSLE; f. the level of platelet; g. drop of hemoglobin; h. C3; i. SLEDAI; j. infection; k. CTX treatment; l. IVIG treatment; m. plasmapheresis; n. mechanical ventilation; NPSLE: neuropsychiatric lupus erythematosus; C3: complement 3; SLEDAI: systemic lupus erythematosus disease activity index; CTX: cyclophosphamide; IVIG: intravenous immunoglobulin. [file 13075_2021_2435_MOESM2_ESM.tif]

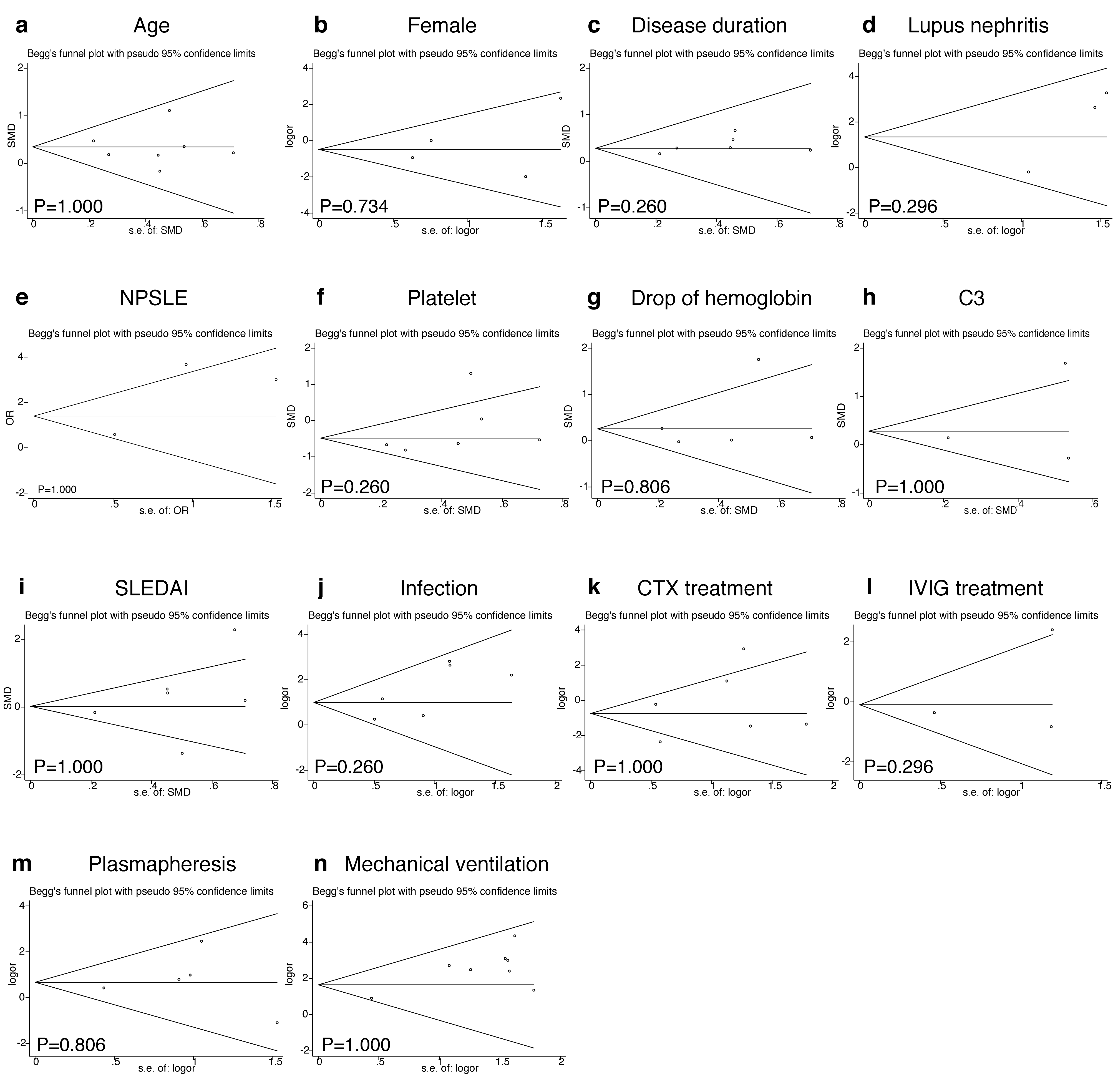

Supplement: Supplementary file 3 — Additional file 3 : Begg’s funnel plots of the publication bias. a. age; b. the female group; c. disease duration. d. lupus nephritis; e. NPSLE; f. the level of platelet; g. drop of hemoglobin; h. C3; i. SLEDAI; j. infection; k. CTX treatment; l. IVIG treatment; m. plasmapheresis; n. mechanical ventilation; NPSLE: neuropsychiatric lupus erythematosus; C3: complement 3; SLEDAI: systemic lupus erythematosus disease activity index; CTX: cyclophosphamide; IVIG: intravenous immunoglobulin. [file 13075_2021_2435_MOESM3_ESM.tif]
